# SpiLinC: Spiking Liquid-Ensemble Computing for Unsupervised Speech and Image Recognition

**DOI:** 10.3389/fnins.2018.00524

**Published:** 2018-08-23

**Authors:** Gopalakrishnan Srinivasan, Priyadarshini Panda, Kaushik Roy

**Affiliations:** Department of ECE, Purdue University, West Lafayette, IN, United States

**Keywords:** spiking neural networks, liquid-ensemble computing, unsupervised multimodal learning, speech recognition, pattern recognition

## Abstract

In this work, we propose a Spiking Neural Network (SNN) consisting of input neurons sparsely connected by plastic synapses to a randomly interlinked liquid, referred to as Liquid-SNN, for unsupervised speech and image recognition. We adapt the strength of the synapses interconnecting the input and liquid using Spike Timing Dependent Plasticity (STDP), which enables the neurons to self-learn a general representation of unique classes of input patterns. The presented unsupervised learning methodology makes it possible to infer the class of a test input directly using the liquid neuronal spiking activity. This is in contrast to standard Liquid State Machines (LSMs) that have fixed synaptic connections between the input and liquid followed by a readout layer (trained in a supervised manner) to extract the liquid states and infer the class of the input patterns. Moreover, the utility of LSMs has primarily been demonstrated for speech recognition. We find that training such LSMs is challenging for complex pattern recognition tasks because of the information loss incurred by using fixed input to liquid synaptic connections. We show that our Liquid-SNN is capable of efficiently recognizing both speech and image patterns by learning the rich temporal information contained in the respective input patterns. However, the need to enlarge the liquid for improving the accuracy introduces scalability challenges and training inefficiencies. We propose SpiLinC that is composed of an ensemble of multiple liquids operating in parallel. We use a “divide and learn” strategy for SpiLinC, where each liquid is trained on a unique segment of the input patterns that causes the neurons to self-learn distinctive input features. SpiLinC effectively recognizes a test pattern by combining the spiking activity of the constituent liquids, each of which identifies characteristic input features. As a result, SpiLinC offers competitive classification accuracy compared to the Liquid-SNN with added sparsity in synaptic connectivity and faster training convergence, both of which lead to improved energy efficiency in neuromorphic hardware implementations. We validate the efficacy of the proposed Liquid-SNN and SpiLinC on the entire digit subset of the TI46 speech corpus and handwritten digits from the MNIST dataset.

## 1. Introduction

SNNs are a class of bio-inspired neuromorphic computing paradigm that closely emulate the organization and computational efficiency of the human brain for complex classification and recognition tasks. Several SNN architectures have been independently proposed for learning visual and auditory signal modalities. Two-layered fully-connected SNN (Diehl and Cook, 2015) and shallow/deep convolutional SNN (Masquelier and Thorpe, 2007; Lee et al., 2016, 2018a,b; Panda and Roy, 2016; Tavanaei et al., 2016; Panda et al., 2017a; Ferré et al., 2018; Jin et al., 2018; Kheradpisheh et al., 2018; Thiele et al., 2018; Wu et al., 2018) have been demonstrated for visual image recognition. On the other hand, reservoir (Lukoševičius and Jaeger, 2009) or liquid computing models (Maass et al., 2002, 2003; Panda and Srinivasa, 2018) have been shown to encode time-varying speech and video data, where subsequent inference entails supervised algorithms. Each class of applications essentially requires separate hardware implementations. Hence, it is highly desirable to have a general computing model capable of processing different signal modalities using a uniform self-learning methodology.

In this work, we propose a general computing model referred to as the Liquid-SNN, consisting of input neurons sparsely connected by plastic synapses to a reservoir of spiking neurons termed *liquid*, for unsupervised learning of both speech and image patterns. The liquid, whose architecture is inspired by neural microcircuits in the cerebral cortex (Maass et al., 2003), consists of excitatory and inhibitory neurons interlinked in a sparse random manner. The recurrent connectivity and the non-linear neuronal dynamics enable the liquid to generate high dimensional spike patterns (liquid states) for varied inputs. Standard liquid computing models (Maass et al., 2002, 2003) have fixed synaptic connectivity between the input and excitatory neurons, which leads to random liquid projections that are extracted by a readout layer. In order to empower the liquid to produce states correlated with the input patterns, we adapt the input to liquid synaptic weights using STDP in our Liquid-SNN. This sensitizes individual excitatory neurons to unique input classes, which facilitates unsupervised learning and inference without a readout layer. The presented learning methodology renders the liquid capable of recognizing time-varying speech inputs as well as static image patterns. Further, we experimentally demonstrate uniform recurrent connectivity across different kinds of applications, which is a testament to the universality of our Liquid-SNN. However, the Liquid-SNN suffers from scalability challenges due to the need to primarily increase the number of neurons to enhance the classification accuracy. To this effect, we propose Spiking Liquid-Ensemble Computing, referred to as *SpiLinC*, which is composed of a distributed arrangement of multiple liquids operating in parallel. SpiLinC incorporates the principle of ensemble learning to recognize an input pattern by training the constituent liquids to extract low-level characteristic features. Thus, SpiLinC is a universal as well as a scalable computing framework that can achieve efficient feature learning.

Liquid State Machine (LSM), a well established liquid computing model, consists of an input layer sparsely connected via synapses whose weights are fixed *a priori* to a liquid followed by a readout layer trained using supervised algorithms (Auer et al., 2002; Verstraeten et al., 2005b) to periodically extract the liquid states and infer the class of an input pattern. We note that unsupervised training of the liquid to readout connections using STDP would increase the network complexity by necessitating larger number of readout neurons with lateral inhibition. Moreover, LSMs have primarily been demonstrated for speech recognition. We find that training such LSMs is challenging for image recognition applications that have sufficient intra-class differences and inter-class similarities. The complex datasets negatively impact the ability of a liquid to produce linearly separable states for distinct input classes. Prior research efforts attempted to improve the liquid efficacy by evolving the recurrent connections (Norton and Ventura, 2010; Yin et al., 2012; Xue et al., 2013; Chrol-Cannon and Jin, 2015; Roy and Basu, 2016; Bellec et al., 2018), which nonetheless require a readout layer. Recently, Panda and Roy (2017) demonstrated a liquid capable of sequence generation (specifically, words) without using a linear readout and relying on the dynamics of the liquid obtained by learning the recurrent connections to perform character-by-character prediction. While our work is complementary to that of Panda and Roy (2017) and prior LSM efforts, we relax the burden of training the recurrent connections and rely on the unique non-linear representations produced by a liquid with fixed recurrent connections to perform recognition.

In an effort to enhance the liquid projections for different input modalities including the speech and image patterns, we train the input to liquid-excitatory synaptic weights in our Liquid-SNN using STDP that enables unsupervised inference without a readout layer. When a time-varying input, for instance, a speech signal is fed to the liquid, the recurrent connectivity causes a group of excitatory neurons to fire. Performing STDP on the interconnecting synaptic weights causes these neurons to learn the temporal dynamics of the presented input, which effectively renders them sensitive to similar inputs. Hence, the excitatory neuronal spiking activity can directly be used for inference. We show that the presented Liquid-SNN, trained in an unsupervised manner on a subset of spoken digits from the TI46 speech corpus (Liberman et al., 1993), achieves comparable accuracy to that provided by LSMs trained using supervised algorithms. In order to further highlight the generality of our proposed model across different application domains, we illustrate how the non-linear liquid dynamics together with STDP can be used to self-learn image patterns. STDP enables the synapses connecting each excitatory neuron to encode a representation of an image pattern. The recurrent inhibition assists in differentiating the receptive field of various excitatory neurons. We demonstrate the efficacy of Liquid-SNN in image recognition by training it to infer handwritten digits from the MNIST dataset (LeCun et al., 1998).

Finally, we present SpiLinC, which is composed of an ensemble of smaller liquids to address the scalability and training inefficiencies posed by a single large Liquid-SNN. Ensemble methods previously proposed in literature (Opitz and Maclin, 1999; Dietterich, 2000) typically entail multiple weak classifiers trained on the same input data, where diversity in the individual classifiers leads to improved inference accuracy (Hansen and Salamon, 1990). In this work, we utilize a “divide and learn” methodology for SpiLinC that is inspired by recent works on ensemble learning in fully-connected SNN trained for MNIST digit recognition (Shim et al., 2016; Panda et al., 2017b). It is important to note that SpiLinC is a sparser universal computing model that is capable of unsupervised multimodal learning. We train each liquid forming the ensemble in parallel on a distinct segment of the input patterns as described in Panda et al. (2017b). The individual liquids consequently learn low-level attributes characterizing different input patterns. SpiLinC thereafter produces inference based on the combined spiking activity of the constituent liquids. Our results indicate that SpiLinC, with sufficient number of neurons per liquid, performs on par with a similarly sized Liquid-SNN. The superior learning efficiency is achieved with improved sparsity and fewer training examples, both of which can be exploited to achieve significant energy savings in custom on-chip implementations like TrueNorth (Merolla et al., 2014) or memristive crossbar architectures (Jo et al., 2010; Rajendran et al., 2013).

Overall, the key contributions of our work are:

1. We propose Liquid-SNN (single liquid architecture) and SpiLinC (liquid-ensemble architecture), where each liquid is trained in parallel on a unique input segment, for unsupervised speech and image recognition.
2. We demonstrate that input subdivision enables the individual liquids in SpiLinC to self-learn diverse input features, which can effectively be combined to recognize an input pattern.
3. We propose optimal strategy to determine the ensemble size and input partition per liquid in the ensemble, and sparsity constraints in the input to liquid and recurrent-liquid synaptic connectivity required to achieve efficient unsupervised learning in SpiLinC.
4. We validate and benchmark the proposed models on the MNIST dataset for image recognition and the entire digit subset of the TI46 speech corpus for speech recognition.

## 2. Materials and methods

### 2.1. Computational model of spiking neuron and synapse

**Figure 1:**
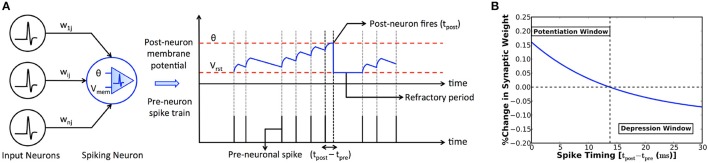
**(A)** SNN consisting of input (pre) neurons connected by weighted synapses (*w*_*ij*_) to a spiking (post) neuron. The input spikes are modulated by the synaptic weights to produce resultant post-synaptic current that leaks exponentially in the time interval between successive input spikes. The spiking neuron integrates the post-synaptic current into its membrane potential (*V*_*mem*_) that subsequently decays in an exponential manner. The neuron fires an output spike (at time instant *t*_*post*_) when its potential exceeds a threshold (θ). The potential is subsequently reset (to *V*_*rst*_) and the neuron is restrained from firing for a certain refractory period. **(B)** Illustration of the power-law weight-dependent STDP rule formulated in (1) for learning rate (η) of 0.005, time constant (τ) of 15ms, *STDP*_*offset*_ of 0.4, maximum weight (*w*_*max*_) of unity, current weight (*w*) of 0.5, and exponential factor (μ) of 0.9. The synaptic weight is increased (potentiated) for strong temporal correlation between a pair of pre- and post-neuronal spikes (*t*_*post*_−*t*_*pre*_ ≤ 14 ms for the chosen parameters), and decreased for larger spike time differences.

The fundamental computing unit of an SNN is a spiking (post) neuron, which is driven by a group of input (pre) neurons via weighted synapses as shown in Figure 1A. The input pre-neuronal spikes are modulated by the synaptic weights to produce resultant post-synaptic current that leaks exponentially in the time interval between successive input spikes. The post-synaptic current is referred to as excitatory (inhibitory) post-synaptic current if the pre-neurons are excitatory (inhibitory). We use the Leaky-Integrate-and-Fire (LIF) model (Diehl and Cook, 2015) to mimic the spiking neuronal dynamics. An LIF neuron integrates the excitatory and inhibitory post-synaptic currents, resulting in a change in its membrane potential that subsequently decays in an exponential manner. It fires an output spike when its potential exceeds an adaptive threshold. The membrane potential is thereafter reset, the firing threshold is raised, and the neuron is prevented from spiking for a certain refractory period. These mechanisms collectively regulate the neuronal spiking activity and help achieve uniform firing rate across neurons in an SNN, thereby facilitating competitive learning.

### 2.2. Synaptic plasticity

Spike Timing Dependent Plasticity (STDP) postulates that the strength (or weight) of a synapse depends on the degree of timing correlation between the corresponding pre- and post-neuronal spikes. We use the power-law weight-dependent STDP model (Diehl and Cook, 2015) that is illustrated in Figure 1B and described by

(1) $$\begin{array}{r} \Delta w=\eta \times \left[e^{-\frac{t_{\text{post}}-t_{\text{pre}}}{\tau }}-{\text{STDP}}_{\text{offset}}\right]\times {\left[w_{\text{max}}-w\right]}^{\mu } \end{array}$$

where Δ*w* is the change in the synaptic weight, η is the learning rate, *t*_*pre*_ and *t*_*post*_ are respectively the time instants of a pair of pre- and post-neuronal spikes, τ is the STDP time constant, *w*_*max*_ is the maximum bound imposed on the synaptic weight, and *w* is the current weight. The synaptic weight is increased (potentiated) if a pre-neuronal spike causes the post-neuron to fire within a definite period of time determined by the *STDP*_*offset*_. On the contrary, the synaptic weight is decreased (depressed) for larger spike time differences. Additionally, the synaptic weight change has an exponential dependence (controlled by μ) on the current weight, which ensures a steady rise (decline) of the synaptic weight toward its upper (lower) bound that is desirable for efficient learning.

### 2.3. Proposed liquid computing models

#### 2.3.1. Single-layered liquid-SNN

**Figure 2:**
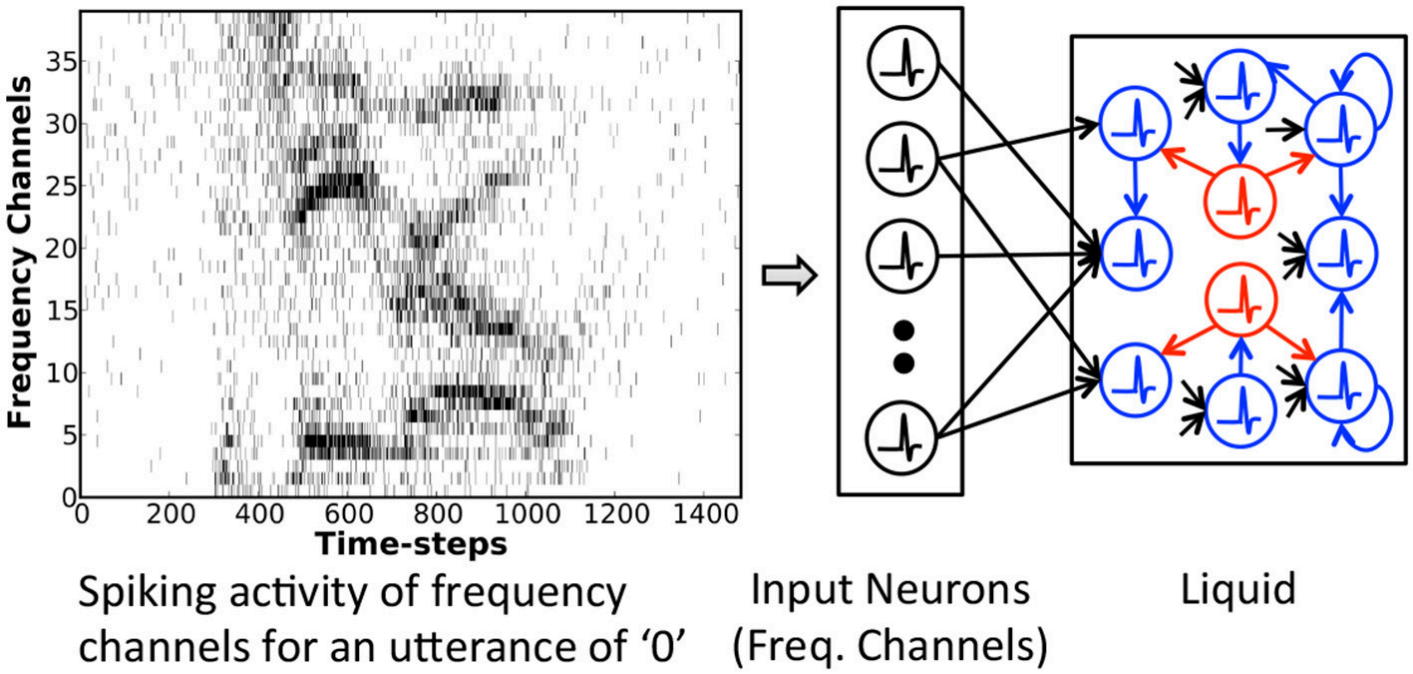
Single-layered Liquid-SNN consisting of input neurons (represented by the time evolution of frequency channels characterizing an utterance of “0” in this sample illustration) sparsely connected to a liquid housing excitatory (highlighted in blue) and inhibitory (highlighted in red) neurons recurrently interlinked in a sparse random manner.

The Liquid-SNN (shown in Figure 2) is composed of an input layer sparsely connected by plastic synapses to a liquid containing excitatory and inhibitory neurons recurrently interlinked in sparse random manner. The excitatory neurons typically outnumber the inhibitory neurons by a factor of 4 as observed in the cortical microcircuits. The neurons in the input layer represent either the time evolution of various frequency components characterizing a speech signal or static image pixels, each firing at a rate proportional to the corresponding intensity. The input neurons are sparsely connected to the excitatory neurons. The non-linear spiking neuronal dynamics together with sparse recurrent connectivity triggers the liquid to produce diverse spiking patterns, referred to as liquid states, for inputs with disparate temporal characteristics. The state of the liquid at any given time is a high dimensional representation incorporating the input dynamics both at the current and preceding time instants. It is important to note that sparsity in synaptic connectivity is essential for a liquid to generate discernible states. A disproportionate increase in the input or recurrent connectivity could potentially result in chaotic spiking activity.

Existing liquid computing models such as LSMs use predetermined weights for the input to liquid synapses and feed the liquid states to a layer of readout neurons for inference. The utility of LSMs has predominantly been demonstrated for speech recognition applications. Image patterns, on the other hand, possess appreciable similarities across classes and marked differences within a class. We observe that complex inputs hinder the capacity of a liquid to produce linearly separable states for different patterns. This results in a loss of information that diminishes the recognition capability of the subsequent readout layer. Previous research investigated self-learning the structure or morphology of the liquid, which enables it to generate specific features rather than random projections. We relax the burden of training the recurrent connections and directly learn the rich temporal information contained in a speech/image pattern by performing STDP on the sparse synaptic connections between the input and liquid. This, in turn, empowers the non-linear liquid (with random recurrent connectivity) to produce states correlated with the input. The proposed methodology effectively tunes the spiking activity of individual neurons to correspond to specific input classes, which enables unsupervised inference without a readout layer as explained below for a speech input. In the beginning of the training phase, a time-varying speech input triggers a random group of excitatory neurons to fire. Carrying out STDP-based updates on the input to liquid synaptic weights allows the neurons to learn temporal correlations underlying the presented input. This increases the likelihood of the corresponding neurons to thereafter spike for similar inputs. The recurrent inhibition facilitates the activation of distinct excitatory neurons for various input classes. STDP imparts temporal learning and sensitizes each neuron to the appropriate input class, which enables the Liquid-SNN to recognize a test input straightaway by using the excitatory neuronal spiking activity as explained below. Once the input to liquid synapses are trained, we tag the excitatory neurons in the liquid with the classes of input patterns for which they spiked at a higher rate during the training phase. During the inference phase, for a given test pattern, we first estimate the average spike count of every group of neurons tagged as having learnt features pertaining to a specific class of input patterns that is specified by

(2) $$\begin{array}{r} avg\text{\_}spike\text{\_}count\left(j\right)=\frac{1}{n_{j}}\overset{n_{j}}{\underset{k=1}{\sum }}spike\text{\_}count\left(k,j\right)\;\forall \;j\in \left\{1\ldots n_{\text{classes}}\right\} \end{array}$$

where *avg*_*spike*_*count*(*j*) is the average spike count of the group of neurons carrying the tag “*j*,” *n*_*j*_ is the total number of neurons with the tag “*j*,” *spike_count*(*k, j*) is the spike count of the *k*^th^ neuron with the tag “*j*” over the time interval for which the test pattern is presented (also referred to as the simulation interval), and *n*_*classes*_ is the number of classes for a given pattern recognition task. We then predict the unknown test pattern to belong to the class (or tag) represented by the neuronal group with the highest average spike count as described by

(3) $$\begin{array}{r} predicted\;test\;class=\underset{j\in \left\{1\ldots n_{\text{classes}}\right\}}{\text{argmax}}avg\text{\_}spike\text{\_}count\left(j\right) \end{array}$$

where *predicted test class* is the class of the test pattern predicted by the Liquid-SNN using the average spike count of the excitatory neurons and the corresponding tags.

In order to illustrate the generality of the proposed model, we describe how the non-linear liquid dynamics together with STDP can be used for the unsupervised learning of image patterns. STDP, when applied to synapses connecting the input to a specific excitatory neuron, causes the synapses to encode a representation of the image pattern in the corresponding weights. This triggers the neuron to fire consistently for matching image patterns while STDP reinforces the synapses. Besides, the positive recurrent connection between pairs of excitatory neurons facilitates them to learn varying representations of a particular class of patterns. On the contrary, the inhibitory connections help separate the receptive field of different excitatory neurons. The proposed topology, in addition to being capable of learning different signal modalities, offers enhanced synaptic sparsity in comparison with two-layered fully-connected SNN (Diehl and Cook, 2015). Despite these advantages, the need to primarily upsize a single liquid for achieving higher classification accuracy introduces scalability issues in hardware realizations.

#### 2.3.2. SpiLinC: spiking liquid-ensemble computing

We present SpiLinC as a scalable neuromorphic computing model composed of a distributed arrangement of multiple liquids, referred to as a *liquid-ensemble*, operating in parallel. An alternative but feasible approach would be to stack smaller liquids in a feed-forward manner to form a deep network. However, the gradual decline in the spiking activity of individual neurons across successive levels of a deep hierarchy limits the effectiveness of STDP on the corresponding synapses. Hence, we propose a liquid-ensemble since it offers inherent parallelism in the operation of its constituent liquids, each of which can be trained efficiently using STDP.

**Figure 3:**
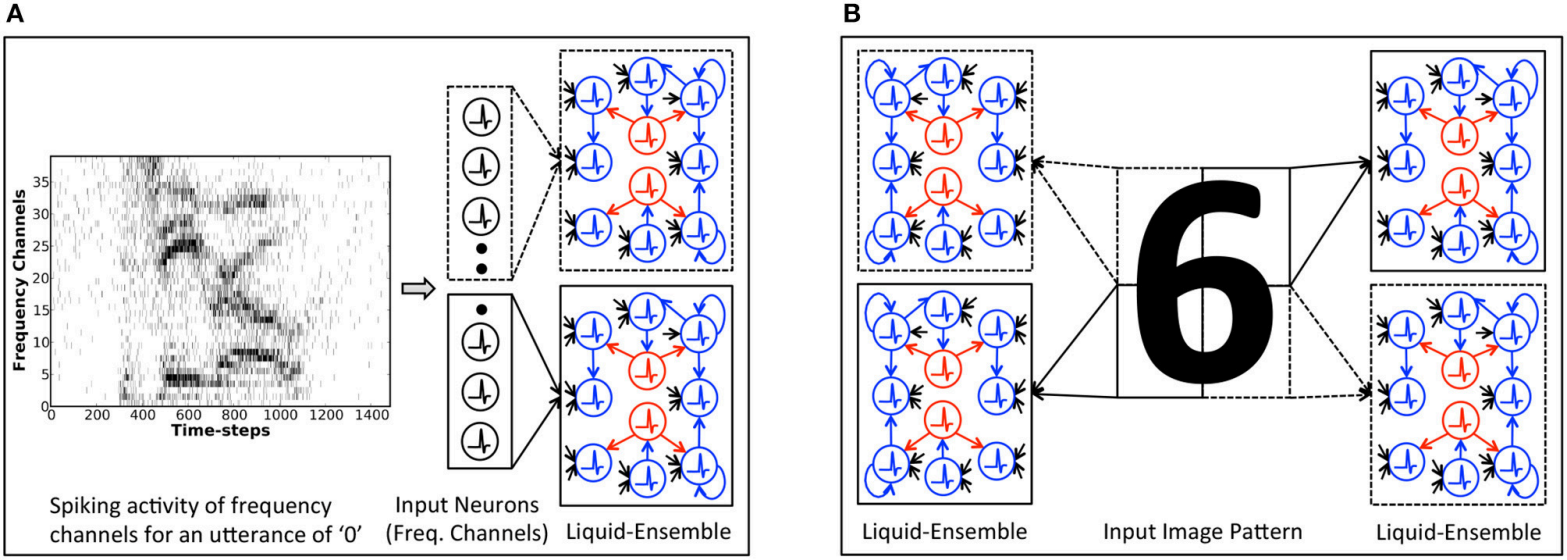
Illustration of the Spiking Liquid-Ensemble Computing (SpiLinC) architecture composed of a distributed arrangement of multiple liquids operating in parallel. **(A)** Two-liquid SpiLinC, where each liquid constituting the ensemble is trained with different frequency channels of a speech signal (utterance of “0” in this example). **(B)** Four-liquid SpiLinC, where the individual liquids are trained with separate partitions of an image pattern.

We adopt a “divide and learn” strategy described by Panda et al. (2017b) for input feature extraction using SpiLinC. Accordingly, we train each liquid in SpiLinC with a distinct input segment. Figure 3A shows a two-liquid SpiLinC architecture trained with different frequency channels of a speech signal while Figure 3B depicts one consisting of four liquids individually trained with separate partitions of an image pattern. Note that there are no synaptic connections between different liquids in SpiLinC. We adapt the strength of the synapses connecting an input partition to a specific liquid using STDP, which enables the excitatory neurons to extract and self-learn distinctive features making up various speech/image patterns. This, in effect, causes each excitatory neuron to spike at a higher rate for those input patterns containing features encoded in the interconnecting synaptic weights. For instance, if the synapses connecting an excitatory neuron encode features contained in the digit pattern “6,” then the corresponding neuron would fire at a higher rate for input patterns similar to “6.” Once all the liquids in SpiLinC are trained, we tag the excitatory neurons in each liquid with the classes of input patterns for which they spiked at a higher rate during the training phase. Since every liquid constituting the ensemble encodes unique input characteristics, SpiLinC integrates the spiking activity of all the liquids to provide a unified decision on the class of a test input. During the inference phase, we present segments of an unknown test pattern to the individual liquids in SpiLinC and record the spike count of all the excitatory neurons. We then estimate the average spike count of every group of neurons (in all the liquids) tagged as having learnt features pertaining to a specific class of input patterns as described by

(4) $$\begin{array}{l} avg\_spike\_count_{i}(j)=\frac{1}{n_{ij}}\overset{n_{ij}}{\underset{k=1}{\sum^{\text{​}}}}spike\_count_{i}(k,j)\\ \;\forall \;i\in \{1\ldots n_{liquids}\}\;and\;j\in \{1\ldots n_{classes}\} \end{array}$$

where *avg*_*spike*_*count*_*i*_(*j*) is the average spike count of the group of neurons in the *i*^th^ liquid carrying the tag “*j*,” *n*_*ij*_ is the total number of neurons in the *i*^th^ liquid with the tag “*j*,” *spike*_*count*_*i*_(*k, j*) is the spike count of the *k*^th^ neuron in the *i*^th^ liquid with the tag “*j*” over the simulation interval, *n*_*liquids*_ is the number of liquids in SpiLinC, and *n*_*classes*_ is the number of classes for a given pattern recognition task. Next, we combine the average spike count of the individual liquids to compute the resultant average spike count of the n-liquid SpiLinC for every possible input class (or tag) that is specified by

(5) $$\begin{array}{r} res\text{\_}avg\text{\_}spike\text{\_}count\left(j\right)=\frac{1}{n_{\text{liquids}}}\overset{n_{\text{liquids}}}{\underset{i=1}{\sum }}avg\text{\_}spike\text{\_}count_{i}\left(j\right) \end{array}$$

where *res*_*avg*_*spike*_*count*(*j*) is the resultant average spike count of the groups of neurons in the n-liquid SpiLinC with the tag “*j*.” Finally, we predict the unknown test pattern to belong to the class (or tag) represented by the neuronal group with the highest resultant average spike count as described by

(6) $$\begin{array}{r} predicted\;test\;class=\underset{j\in \left\{1\ldots n_{\text{classes}}\right\}}{\text{argmax}}res\text{\_}avg\text{\_}spike\text{\_}count\left(j\right) \end{array}$$

where *predicted test class* is the class of the test pattern predicted by SpiLinC. Thus, we combine the spiking activity of each liquid that separately learnt characteristic input features to make a strong prediction regarding the class of a test pattern. SpiLinC effectively incorporates STDP-driven ensemble learning mechanism to provide a universal and scalable liquid computing model capable of unsupervised speech and image recognition.

We now provide insights on the sparsity and training efficiency of SpiLinC against an equivalently sized Liquid-SNN. Given a fixed number of training examples, SpiLinC is inherently capable of encoding more input information than the Liquid-SNN. In other words, the Liquid-SNN requires greater number of training examples to attain similar efficiency in learning. This is corroborated by our analysis, which reveals that SpiLinC with adequate number of neurons per liquid performs on par with the Liquid-SNN using significantly lower number of training examples. SpiLinC, by construction, offers enhanced sparsity than the Liquid-SNN. The favorable trade-offs render SpiLinC more amenable for realizing intelligent devices capable of adapting real-time to varied input data.

### 2.4. Experimental framework

We evaluated the proposed liquid computing models using BRIAN (Goodman and Brette, 2008), which is a Python-based SNN simulator, on the entire digit subset of the TI46 speech corpus and the MNIST handwritten digit dataset. In the following sub-sections, we describe the procedure followed to convert the input speech/image patterns to Poisson-distributed spike trains and generate the Liquid-SNN and SpiLinC topologies, the training and testing methodology, and the metrics used to compare the proposed models.

#### 2.4.1. Input spike generation

We first describe the process of converting the input image patterns to Poisson-distributed spike trains, and then detail the corresponding mechanism for a speech input. For the handwritten MNIST digits, we converted the input pixel intensities to average Poisson firing rates constrained between 0 and 63.75 spikes per second. The average Poisson firing rate of an input neuron is then used to estimate its firing probability (*p*_*firing*_) at every simulation time-step that is specified by

(7) $$\begin{array}{r} p_{firing}=\frac{average\;firing\;rate}{1000}\times t_{step} \end{array}$$

where *t*_*step*_ is the simulation time-step (0.5 ms used in this work). For instance, if the intensity of an image pixel is 255, the average Poisson firing rate for the corresponding input neuron is 63.75 spikes per second and its firing probability at every time-step is 0.031875. We then generate a random number between 0 and 1 at every simulation time-step and emit a spike if the random number is less than the probability of firing. On the other hand, for the TI46 speech dataset, we pre-processed the audio samples available in wave format (.wav extension) based on Lyon's Passive Ear model (Lyon, 1982) of the human cochlea using Slaney's MATLAB auditory toolbox (Slaney, 1998). Using Lyon's cochlear model, which extracts the time evolution of frequency channels characterizing a speech signal, we converted each audio sample into variation in the intensity of 39 frequency channels over time. We then normalized the intensity of all the frequency channels at every time-step with respect to the maximum intensity to map it to the instantaneous firing probability of the corresponding input neurons. Finally, we used Poisson process (described above) to generate the input spikes at every time-step.

#### 2.4.2. Network generation

In this sub-section, we detail the procedure followed to generate the Liquid-SNN and SpiLinC for different pattern recognition tasks. For the Liquid-SNN, the entire input is sparsely connected to the single liquid layer. On the other hand, for SpiLinC, we determined the optimal number of liquids based on the dimensionality of the input data and partitioned the input for the individual liquids as discussed in section 4.3. We then estimated the percentage of input to liquid and recurrent-liquid (excitatory/inhibitory ↔ excitatory/inhibitory) synaptic connections (listed in Table 1) based on the heuristics discussed in section 4.1 on liquid sparsity. In order to generate the connectivity matrix between any two groups of neurons from the percentage of synaptic connections, we first formed a random matrix of dimension, number of neurons in the first group × number of neurons in the second group, and populated it with random numbers following a uniform distribution between 0 and 1. We then created a connection between those pairs of neurons, where the matrix entry is less than the required percentage of synaptic connections divided by 100. For instance, for a liquid with 100 excitatory neurons and 1% connectivity among them, we form a random matrix of dimension, 100 × 100, populate it with random numbers from a uniform distribution between 0 and 1, and create a connection between those pairs of neurons where the random entry is less than 0.01. The input to liquid connections are subjected to synaptic plasticity and hence the corresponding weights are initialized with random values following a uniform distribution between *w*_*init*_*min*_ and *w*_*init*_*max*_ that are listed in Table 2 for different pattern recognition tasks. On the other hand, the recurrent-liquid synaptic connections have constant weight as shown in Table 2. The excitatory and inhibitory neurons making up the liquid are modeled using differential equations mimicking the leaky-integrate-and-fire dynamics as described in Diehl and Cook (2015). The LIF neuronal parameters are adopted from Jug (2012) with the exception of the time constant of the excitatory and inhibitory post-synaptic currents, which have been changed to 2 ms (from 1 ms) and 1 ms (from 2 ms), respectively for enabling the excitatory neurons to spike at a higher rate only during the testing phase.

**Table 1:** Synaptic connectivity parameters.

**Application**	***n*_*e*_/*n*_*i*_**	**Input to liquid connections (*p*_*inp*−*e*_)**	**Recurrent-liquid connections**			
			***p*_*ee*_ (%)**	***p*_*ei*_ (%)**	***p*_*ie*_ (%)**	***p*_*ii*_ (%)**
Speech recognition	3/1	25% (Liquid-SNN) /	0.5	5.0	20.0	0.5
		25% (SpiLinC)				
Image recognition	4/1	30% (Liquid-SNN) /	1.0	5.0	30.0	1.0
		50% (SpiLinC)				

**Table 2:** Synaptic weight initialization parameters.

**Application**	**Input to liquid weights**	**Recurrent-liquid weights**			
		**ee**	**ei**	**ie**	**ii**
Speech recognition	[0.005, 0.505]	1.00	3.00	1.00	1.00
Image recognition	[0.003, 0.303]	1.00	10.0	1.00	1.00

#### 2.4.3. Training and testing framework

During the training phase, each input speech/image pattern is converted to spike trains and fed to the networks for a certain simulation interval, which is chosen to be 750 ms for the TI46 speech samples and 350 ms for the MNIST digits. We used a simulation time-step of 0.5 ms. The synaptic weights connecting the input to each liquid-excitatory neuron are modified at the time instants of an output spike using STDP as mandated by the presented unsupervised learning methodology. Once the networks are trained, each excitatory neuron is tagged with the class of input patterns for which it spiked the most during the training phase for the MNIST digit recognition task. On the other hand, for the TI46 speech recognition task, each excitatory neuron is tagged with two classes of input patterns for which it spiked at a higher rate than the rest of the input classes during the training phase. During the inference phase, the spike count and the tag(s) of the excitatory neurons are used to predict the class of a test pattern as described in sections 2.3.1 (for the Liquid-SNN) and 2.3.2 (for SpiLinC).

#### 2.4.4. Evaluation metrics

We use the classification accuracy (on the testing dataset), the number of input to liquid and recurrent-liquid synapses, and the number of training examples needed for convergence as the evaluation metrics for comparing the proposed Liquid-SNN and SpiLinC topologies. We report the classification accuracy of the networks across five different training and testing runs to account for the randomness in the input to liquid connectivity, the recurrent-liquid connectivity, and the input spike generation scheme based on Poisson process. The evaluation metrics, namely, the number of synapses and training examples, determine the degree of energy efficiency offered by the networks in neuromorphic hardware implementations. The total number of synapses in a network of given size determines the area footprint and read/write energy of the synaptic memory in neuromorphic hardware implementations. The number of synapses in the Liquid-SNN (*#synapses*) is calculated as

(8) $$\begin{array}{l} \#synapses=\frac{p_{inp-e}}{100}\times n_{inp}\times n_{e}+\frac{p_{ee}}{100}\times n_{e}\times n_{e}\\ \;+\frac{p_{ei}}{100}\times n_{e}\times n_{i}+\frac{p_{ie}}{100}\times n_{i}\times n_{e}\\ \;\begin{array}{c} +\frac{p_{ii}}{100}\times n_{i}\times n_{i} \end{array} \end{array}$$

where *n*_*inp*_ is the number of input neurons, *n*_*e*_ is the number of excitatory neurons, *n*_*i*_ is the number of inhibitory neurons, *p*_*inp*−*e*_ is the percentage of synaptic connections between the input and excitatory neurons, *p*_*ee*_ is the percentage of recurrent synaptic connections among the excitatory neurons, *p*_*ei*_ is the percentage of synaptic connections between the excitatory and inhibitory neurons, *p*_*ie*_ is the percentage of synaptic connections between the inhibitory and the excitatory neurons, and *p*_*ii*_ is the percentage of recurrent synaptic connections among the inhibitory neurons. The number of synapses in SpiLinC is estimated by multiplying the number of synapses per liquid [*#synapses* in (8)] with the total number of liquids. We define the sparsity in synaptic connectivity offered by SpiLinC over a similarly sized Liquid-SNN as the ratio of the number of synapses in the Liquid-SNN to the number of synapses in SpiLinC. The final metric, i.e., the number of training examples needed for convergence, which depends on the liquid size (or the number of neurons per liquid), is determined experimentally by increasing the number of training examples until the classification accuracy (on the testing set) attains a maximum and saturates/deteriorates for any further increase in the training examples. For every two-fold increase in the liquid size, we find that the required number of training examples scales by a factor of 2 × to 4 × for the liquid sizes presented in this work across different pattern recognition tasks. The number of training examples dictates the total energy expended during the training phase including the neuronal computational energy, the read energy for loading the input to liquid and the recurrent-liquid synaptic weights every time-step, and the total number of weight updates carried out on the plastic input to liquid synapses that impacts the synaptic write energy. We use the same learning rate for both the networks to perform a fair comparison with respect to the number of training examples needed for convergence.

## 3. Results

### 3.1. Speech recognition

We employ the entire digit subset of the TI46 speech corpus (Liberman et al., 1993) to demonstrate the utility of the proposed Liquid-SNN and SpiLinC in unsupervised speech recognition. The digit subset contains a total of 1,594 training samples including 10 utterances each of digits 0–9 spoken by 16 different speakers and 2542 test samples. Each speech sample is uniquely represented by the time evolution of 39 frequency channels that constitute the input neurons. The parameters governing the input to liquid connectivity, the recurrent-liquid connectivity, and the ratio of the number of excitatory to inhibitory neurons are shown in Table 1 while the STDP model parameters are listed in Table 3.

**Table 3:** STDP model parameters for different pattern recognition tasks.

**Parameters**	**Values**	
	**Speech recognition**	**Image recognition**
learning rate, η	0.0001	0.005
STDP time constant, τ	15 ms	15 ms
STDP offset, *STDP*_**offset**_	0.0	0.4
Weight dependence factor, μ	0.9	0.9
Maximum synaptic weight, *w*_**max**_	1.0	1.0
Minimum synaptic weight, *w*_**min**_	0.0	0.0

**Figure 4:**
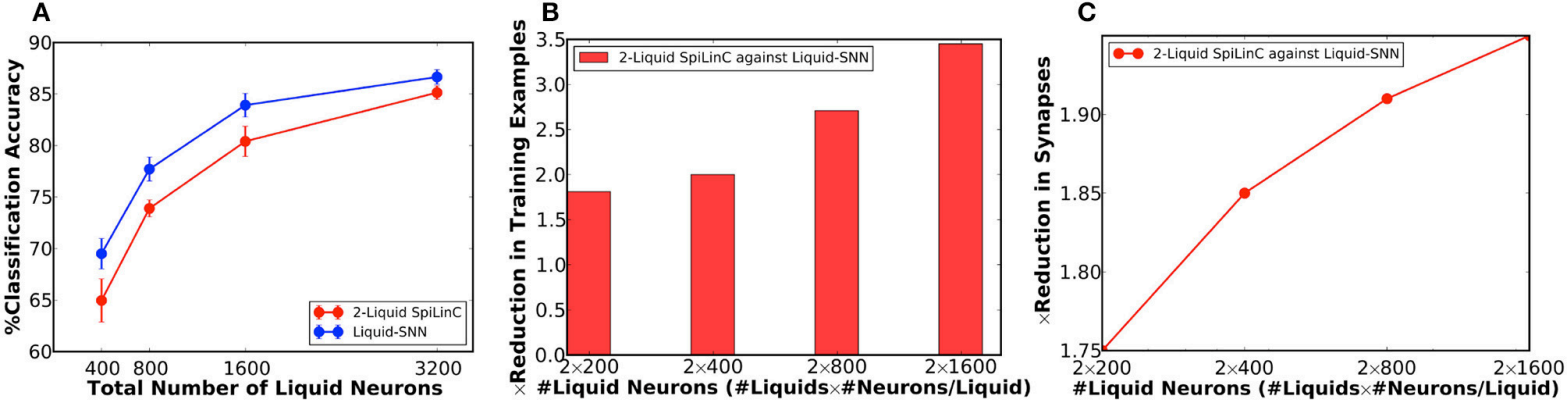
**(A)** Classification accuracy of two-liquid SpiLinC and Liquid-SNN vs. the total number of liquid neurons, evaluated on 2,542 test samples from the TI46 digit subset. **(B)** Reduction in the number of training examples offered by two-liquid SpiLinC over Liquid-SNN. **(C)** Sparsity in synaptic connectivity offered by two-liquid SpiLinC over Liquid-SNN.

Our analysis on a Liquid-SNN of 800 neurons yielded an average accuracy of 77.7%, which signifies the capability of the liquid to self-learn digit utterances. The accuracy increased to 86.66% by augmenting the size of the Liquid-SNN to 3,200 neurons. However, the need to continually grow a single-layered Liquid-SNN for improving the accuracy leads to increased synaptic connectivity and slower training convergence. We present two-liquid SpiLinC as a scalable architecture for achieving improved accuracy. Each liquid in the ensemble is trained on a distinct group of 30 input neurons that enables it to learn the temporal dynamics of the corresponding frequency channels. Given a certain number of training examples, SpiLinC encodes more input information than a similarly sized Liquid-SNN. As a result, SpiLinC achieves efficient feature learning using fewer training examples as corroborated by Table 4 that shows the number of examples used to train both the Liquid-SNN and SpiLinC for different network sizes. Note that the unique input samples are replicated to generate the required number of training examples for different network sizes. Our simulations reveal that the individual liquids need to be suitably sized to match the accuracy of an equivalent Liquid-SNN. This is evidenced by Figure 4A, which indicates that the two-liquid SpiLinC requires a minimum of 1,600 neurons per liquid to attain an accuracy of 85.14%, which is on par with that provided by a Liquid-SNN of 3,200 neurons. The comparable accuracy is achieved with 3.45 × reduction in the number of training examples (as shown in Figure 4B) and 1.95 × sparsity in synaptic connectivity (as depicted in Figure 4C). It can additionally be inferred from Figure 4C that larger the number of liquid neurons, greater is the sparsity offered by SpiLinC over Liquid-SNN.

**Table 4:** Number of examples used to train the Liquid-SNN and SpiLinC for recognizing digit utterances from the TI46 speech corpus.

**#Liquid neurons**	**Number of training examples**	
	**Liquid-SNN**	**Two-liquid SpiLinC**
400	3, 800	2, 100
800	8, 200	4, 100
1,600	26, 000	9, 600
3,200	100, 000	29, 000

**Figure 5:**
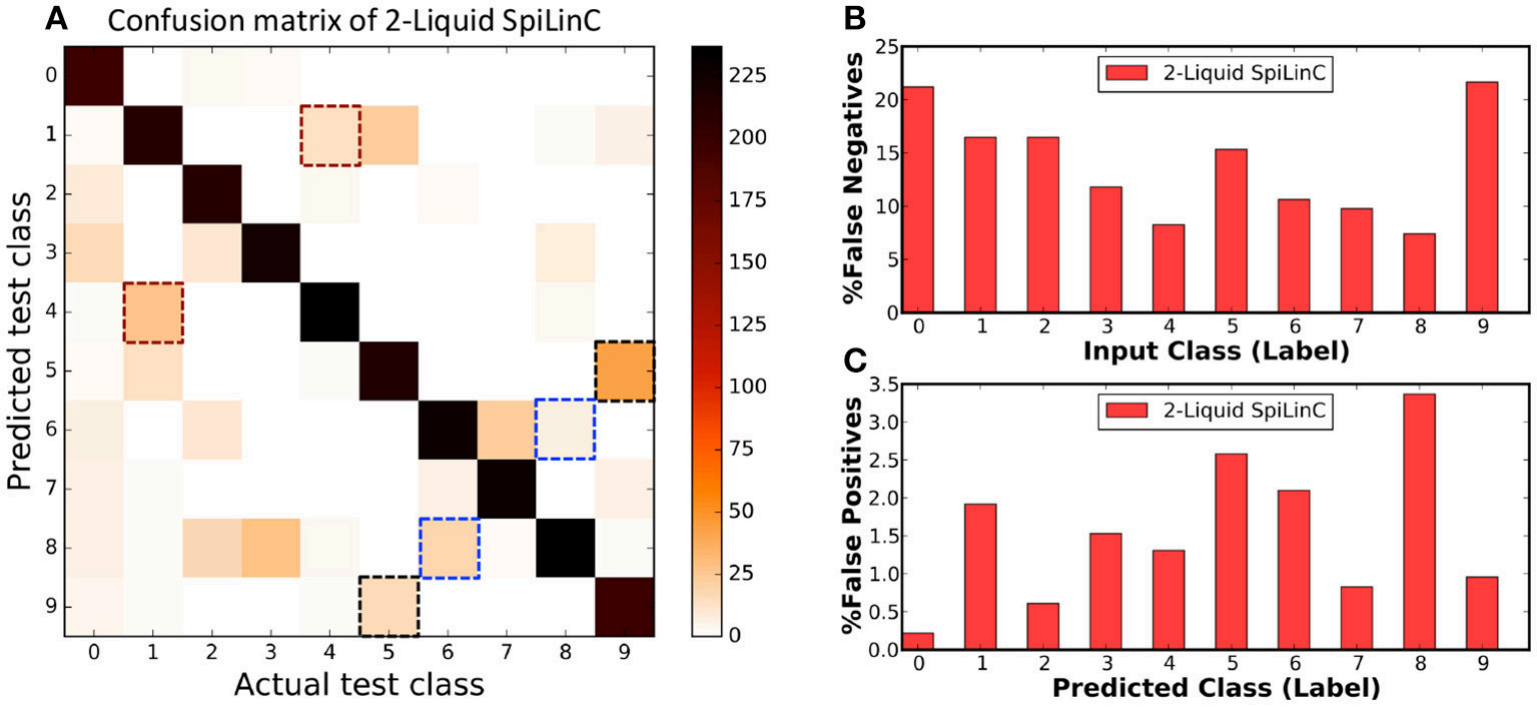
**(A)** Confusion matrix of two-liquid SpiLinC with 1,600 neurons per liquid trained on 29,000 digit utterances from the TI46 speech corpus. **(B)** Percentage of false negatives predicted by the two-liquid SpiLinC for every input class. **(C)** Percentage of false positives produced by the two-liquid SpiLinC for every predicted class.

Finally, we use the confusion matrix illustrated in Figure 5A to dissect the classification performance of the two-liquid SpiLinC with 1,600 neurons per liquid. The confusion matrix for a given network, which plots the predicted class vs. the actual class of the test samples, precisely specifies the number of correct predictions and mispredictions (false negatives and false positives) for every input class. The diagonal elements of the confusion matrix indicate the number of correct network predictions for each input class as depicted in Figure 5A. The false negative misprediction represents the scenario, where the network predicts an incorrect class (for instance, “1–9”) for test input belonging to a different class (for instance, “0”). Figure 5A shows that (“1,” “4”), (“5,” “9”), and (“6,” “8”) are some of the common pairs of input classes misclassified by the two-liquid SpiLinC. The total number of false negatives per input class is estimated by column-wise summation of the confusion matrix excluding the diagonal entry. The percentage of false negatives per input class (plotted in Figure 5B) is determined by dividing the number of false negatives by the total number of test samples in the respective class. We find that the percentage of false negatives predicted by the two-liquid SpiLinC is relatively high (>15%) for input classes “0,” “1,” “2,” “5,” and “9” compared to the remaining classes. On the contrary, the false positive misprediction represents the scenario, where the network predicts a particular incorrect class (for instance, “0”) for test input belonging to any of the remaining classes (for instance, “1–9”). The number of false positives per predicted class is computed by row-wise summation of the confusion matrix excluding the diagonal entry. The percentage of false positives per predicted class is obtained by dividing the number of false positives by the total number of test samples excluding the ones belonging to the predicted class. Our results plotted in Figure 5C indicate that the percentage of false positives is relatively high (>~2%) for predicted classes “1,” “5,” “6,” and “8” compared to the rest of the classes.

### 3.2. Handwritten digit recognition

We demonstrate the applicability of Liquid-SNN and SpiLinC in unsupervised image recognition by training them to infer handwritten digits from the MNIST dataset (LeCun et al., 1998). We use the entire training set containing 60,000 images and report the classification accuracy on the testing set of 10,000 images. Each digit pattern is 28 × 28 in dimension, essentially giving rise to 784 input neurons. It is evident from Table 1 that the recurrent-liquid connectivity is uniform across image and speech recognition applications, which further underlines the generality of the proposed liquid computing models.

**Figure 6:**
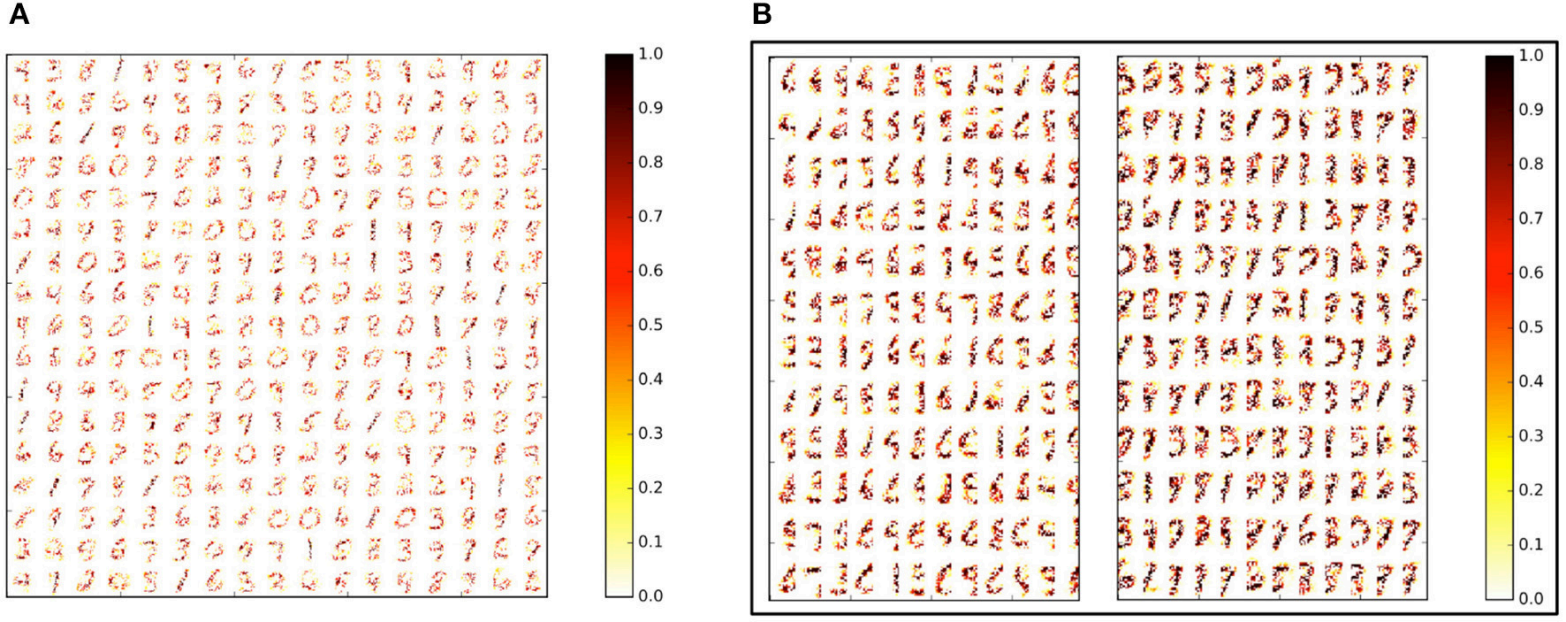
**(A)** Sparse representations of MNIST digits (28 × 28 in dimension) self-learned by a Liquid-SNN of 400 neurons (80% of which are excitatory) in the weight of the synapses connecting the input to the liquid-excitatory neurons (320 excitatory neurons organized in a 17 × 17 grid). **(B)** Sparse features self-learned by the individual liquids making up a two-liquid SpiLinC consisting of 200 neurons per liquid (160 excitatory neurons organized in a 12 × 12 grid) trained using the vertical partitions (each 28 × 16 in dimension) of the MNIST digit patterns.

**Figure 7:**
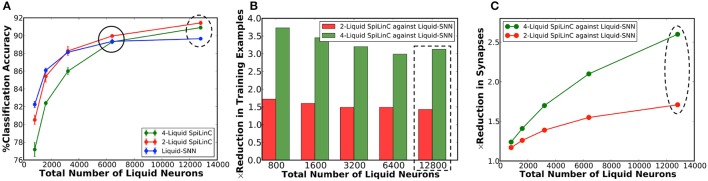
**(A)** Classification accuracy of two-liquid SpiLinC, four-liquid SpiLinC, and Liquid-SNN vs. the total number of liquid neurons, evaluated on the MNIST testing dataset. **(B)** Reduction in the number of training examples offered by two-liquid SpiLinC and four-liquid SpiLinC over Liquid-SNN. **(C)** Sparsity in synaptic connectivity offered by two-liquid SpiLinC and four-liquid SpiLinC over Liquid-SNN.

**Figure 8:**
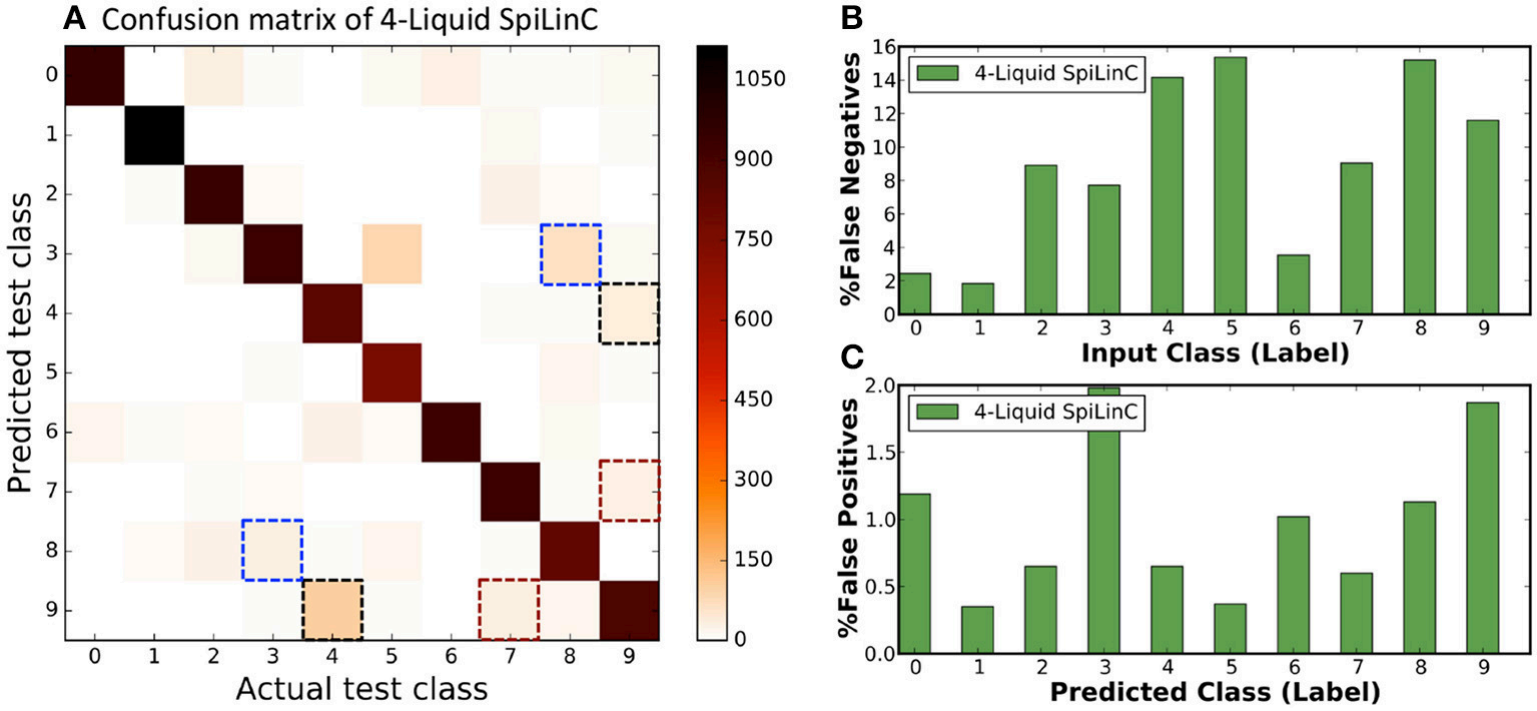
**(A)** Confusion matrix of four-liquid SpiLinC with 3,200 neurons per liquid trained on 64,000 MNIST training examples. **(B)** Percentage of false negatives predicted by the four-liquid SpiLinC for every input class. **(C)** Percentage of false positives produced by the four-liquid SpiLinC for every predicted class.

In our first experiment, we trained a Liquid-SNN of 400 neurons on a smaller subset of training examples (refer to Table 5) using the STDP model parameters shown in Table 3, which yielded an average accuracy of 76.86%. It can be seen from Figure 6A that the synapses connecting every excitatory neuron acquired a sparse representation of a distinct handwritten digit. This illustrates the capacity of the non-linear liquid to self-learn image patterns, which is enabled by the presented unsupervised learning methodology. We obtained an improved accuracy of 89.65% using an augmented liquid of 12,800 neurons. In an effort to achieve further improvements in accuracy with added sparsity benefits, we explored a couple of liquid-ensemble schemes, namely, two-liquid SpiLinC and four-liquid SpiLinC. The two-liquid SpiLinC is trained using the vertical image partitions (each 28 × 16 in dimension) while the four-liquid SpiLinC is additionally trained using the horizontal image partitions (each 16 × 28 in dimension). Figure 6B illustrates the characteristic features self-learned by two-liquid SpiLinC consisting of 200 neurons per liquid. However, the resultant accuracy (75.37%) is lower than that attained by a Liquid-SNN of 400 neurons (76.86%). This is because the individual liquids need to be sufficiently sized for them to comprehensively learn various distinctive features. This is validated by Figure 7A, which shows that two-liquid SpiLinC with 3,200 (>3,200) neurons per liquid performs on par with (better than) an equivalently sized Liquid-SNN. Further, two-liquid SpiLinC with 6,400 neurons per liquid provides an improved accuracy of 91.43%, which is achieved with 1.4 × fewer training examples (Figure 7B) and 1.7 × sparsity (Figure 7C) compared to the Liquid-SNN. Similar trends are observed for four-liquid SpiLinC, which necessitates 1,600 (>1,600) neurons per liquid to attain comparable (higher) accuracy than the Liquid-SNN. Four-liquid SpiLinC with 3,200 neurons per liquid offers an accuracy of 90.9%, which is achieved with 3 × fewer training examples and 2.6 × sparsity than the Liquid-SNN. Hence, the four-liquid SpiLinC with 3,200 neurons per liquid yields the best trade-off among accuracy, sparsity, and training convergence for MNIST digit recognition. Figure 8A illustrates the confusion matrix of the four-liquid SpiLinC with 3,200 neurons per liquid. We find that the four-liquid SpiLinC frequently misclassifies certain pairs of input classes including (“3,” “8”), (“4,” “9”), and (“7,” “9”), where the individual classes in the respective pairs share common features. Overall, the four-liquid SpiLinC predicts a higher percentage (>10%) of false negatives for the input classes “4,” “5,” “8,” and “9” as evidenced by Figure 8B. On the other hand, the percentage of false positives (plotted in Figure 8C) is higher for the predicted classes “3” and “9” compared to the rest of the classes.

**Table 5:** Number of examples used to train the Liquid-SNN and SpiLinC for recognizing MNIST digits.

**#Liquid neurons**	**Number of training examples**		
	**Liquid-SNN**	**Two-liquid SpiLinC**	**Four-liquid SpiLinC**
400	5,500	3,000	–
800	11,200	6,500	3,000
1,600	22,400	14,000	6,500
3,200	44,800	30,000	14,000
6,400	89,600	60,000	30,000
12,800	200,000	140,000	64,000

Our experimental results on SpiLinC (compared to Liquid-SNN) including the number of training examples listed in Table 5 (normalized plot in Figure 7B) and the reduction in the number of synapses (Figure 7C), respectively indicate that higher the number of liquids, faster is the training convergence and greater is the sparsity in synaptic connectivity. However, scaling up the number of liquids (ensemble size) lowers the size of the input segment received by the individual liquids. This limits the feature learning capability of SpiLinC, leading to degradation in the classification accuracy. The optimal ensemble size was experimentally determined to be 4 for MNIST digit recognition. Refer to section 4.3 for a discussion on the scalability of SpiLinC across different tasks.

## 4. Discussion

### 4.1. Liquid sparsity

The proposed liquid computing models necessitate sparsity in both the input to liquid and recurrent-liquid synaptic connectivity to achieve efficient unsupervised learning. In our experiments using Liquid-SNN, we enforced 25% and 30% synaptic connections between the input and liquid-excitatory neurons, respectively for the speech and image recognition applications. Note that SpiLinC could have a higher percentage of input to liquid synaptic connections as shown in Table 1 for the image recognition task since the individual liquids receive only a segment of overall input. However, the total number of synaptic connections (input to liquid) is similar for both SpiLinC and Liquid-SNN. The sparsity in the input to liquid synaptic connectivity prevents hyperactivity of the liquid neurons, which is essential for self-learning input representations (using Liquid-SNN) or characteristic features (using SpiLinC). It is interesting to note that the sparsity in the input to liquid synaptic connectivity, which is defined as the ratio of the total number of synaptic connections to the effective number of synaptic connections, increases during training as STDP prunes the insignificant synaptic connections like those from the background pixels of the MNIST digit patterns. We quantify the change in sparsity for MNIST digit recognition using four-liquid SpiLinC containing 3,200 neurons per liquid (out of which 2,560 are excitatory), where each liquid receives input from 28 × 16 image segment. The total number of input to liquid synaptic connections is 1,146,880 (28 × 16 × 2,560). Before training, the effective number of input to liquid synaptic connections is initialized to 50% of the total number of synaptic connections as shown in Table 1, leading to 2 × sparsity. During training, STDP reduced the effective number of input to liquid synaptic connections to an average of 230,786 per liquid, thereby increasing the sparsity to 4.96 ×. For TI46 speech recognition, the sparsity in the input to liquid synaptic connectivity remains the same during training since the optimal STDP parameters (listed in Table 3) ensure synaptic weights are never depressed.

Next, we discuss the sparsity requirement in the recurrent connectivity within the liquid (individual liquids) constituting Liquid-SNN (SpiLinC). Note that SpiLinC, by construction, offers enhanced sparsity in recurrent connectivity by breaking up a single large Liquid-SNN into an ensemble of multiple liquids operating in parallel. Our analysis on both Liquid-SNN and SpiLinC indicate that it is desirable to have limited synaptic connectivity between pairs of excitatory (*ee* connections) or inhibitory neurons (*ii* connections). We find that excessive *ee* and *ii* synaptic connections lead to disproportionate increase in the spiking activity of the excitatory neurons while lowering the efficacy of the inhibitory neurons. This results in chaotic spiking activity within the liquid that is detrimental to learning. In this work, we used 0.5–1% *ee* and *ii* synaptic connections. However, we introduced a relatively higher percentage of synaptic connections (5%) between the excitatory and inhibitory neurons (*ei* connections) to sufficiently excite the inhibitory neurons. These neurons inhibit and help differentiate the receptive filed of various excitatory neurons. Hence, the inhibitory to excitatory (*ie*) synaptic connections ought to be substantial (20–30% used in this work) for enabling the liquid(s) to self-learn varied inputs in a multi-class pattern recognition task. It is important to note that the relative sparsity among the input to liquid and recurrent-liquid (*ee*, *ei*, *ie*, and *ii*) connectivity controls the spiking activity within the liquid, which is critical for improved representation/feature learning and achieving competitive classification accuracy. The sparsity in the recurrent-liquid synaptic connectivity remains the same during training since the connectivity matrices are fixed *a priori*.

### 4.2. SpiLinC training convergence

We comprehensively validated the capability of SpiLinC to achieve faster training convergence (using fewer examples) compared to Liquid-SNN across speech (Table 4) and image recognition tasks (Table 5). It is important to note that SpiLinC exploits the principle of ensemble learning to achieve faster training convergence as illustrated below. Let us suppose that a Liquid-SNN containing *x* neurons requires a total of *y* training examples to yield the optimal classification accuracy. Intuitively, a larger liquid with 2*x* neurons, which necessitates a minimum of 2*y* training examples, can be used to improve the classification accuracy. Alternatively, consider a two-liquid SpiLinC (with *x* neurons per liquid) instead of a single large Liquid-SNN (with 2*x* neurons). The individual liquids can effectively be trained in parallel using *y* (>*y* in practice) training examples. We showed that training the constituent liquids on disparate input segments causes them to self-learn characteristic input features. SpiLinC subsequently infers the class of a test input by identifying the underlying low-level features. This enables the two-liquid SpiLinC to perform on par or even outperform the Liquid-SNN (beyond certain value of *x*) using 2 × (< 2 × in practice) fewer training examples. Higher the number of liquids forming SpiLinC, greater is the reduction in the number of training examples and faster is the convergence compared to Liquid-SNN. This is corroborated by our experimental results, which indicate that two-liquid SpiLinC, on average, learns with 1.95 × fewer training examples while four-liquid SpiLinC offers up to 3.3 × reduction in the number of training examples.

### 4.3. SpiLinC scalability

The scalability of SpiLinC with respect to the number of liquids (ensemble size) determines the trade-off between classification accuracy on one hand and sparsity together with training convergence on the other for a given task. Scaling up the number of liquids, in general, leads to improved sparsity and faster training convergence (as discussed in sections 4.1 and 4.2). However, the classification accuracy is comparable to (better than) that provided by Liquid-SNN only up to a certain ensemble size. The accuracy deteriorates rapidly for larger ensembles due to insufficient inputs received by the liquids. The number of inputs received by each liquid in SpiLinC is specified by

(9) $$\begin{array}{r} Numberofinputsperliquid\\ \;=p_{inp-e}\times \left[\frac{inputdimension}{n_{\text{liquids}}}+n_{overlap}\right] \end{array}$$

where *p*_*inp*−*e*_ specifies the %synaptic connectivity between the input and liquid, *inputdimension* is the overall dimension of the input data, and *n*_*overlap*_ is the number of overlapping inputs across ensembles. It is evident from (9) that the effective number of inputs per liquid decreases for larger ensembles (*n*_*liquids*_). This has a detrimental impact on the feature learning efficacy of the individual liquids, leading to degradation in the classification accuracy. Hence, the optimal ensemble size for a given task, which depends on the input dimension as shown in (9), needs to be selected so as to guarantee adequate number of inputs to each liquid. For the speech recognition task with a total of 39 frequency channels representing each speech sample, two-liquid SpiLinC (≥1,600 neurons per liquid) receiving inputs from 30 disparate frequency channels was determined to be the optimal configuration. On the other hand, we demonstrated up to four-liquid SpiLinC (≥1,600 neurons per liquid) for efficiently recognizing MNIST digit patterns, 28 × 28 in dimension.

### 4.4. Comparison with related works

We compare the proposed Liquid-SNN and SpiLinC models with the LSM presented in Verstraeten et al. (2005a) that uses a similar pre-processing front-end for the TI46 speech recognition task. We use a smaller subset containing a total of 500 speech samples that includes 10 utterances each of digits 0–9 spoken by 5 different female speakers since it is de facto used in existing works to evaluate models for speech recognition. We trained our models on 300 randomly selected speech samples and report the classification accuracy on the remaining 200 samples. Table 6 shows that both the Liquid-SNN and two-liquid SpiLinC yield comparable albeit slightly lower classification accuracy than that provided by the LSM, which requires a readout layer trained in a supervised manner to infer the class of an input pattern. Next, we evaluate the proposed models against a two-layered fully-connected SNN (Diehl and Cook, 2015) that is commonly used for unsupervised image recognition. Table 7 shows that the classification accuracy of both the models on the MNIST testing dataset is lower than an accuracy of 95% achieved by the two-layered SNN. However, the Liquid-SNN (with 12,800 neurons) and SpiLinC (4 × 3,200 neurons), respectively offer 3.6 × and 9.4 × sparsity in synaptic connectivity compared to the two-layered SNN containing 6,400 excitatory and 6,400 inhibitory neurons. Note that number of synapses of the baseline two-layered SNN (shown in Table 7) is computed from (8) using the following parameters: *n*_*inp*_ = 784, *n*_*e*_ = *n*_*i*_ = 6,400, *p*_*inp*−*e*_ = 1, *p*_*ee*_ = *p*_*ii*_ = 0, *p*_*ei*_ = 1/*n*_*e*_, and *p*_*ie*_ = 1−*p*_*ei*_. Further, the four-liquid SpiLinC with 3,200 neurons per liquid (out of which 2,560 neurons are excitatory) would converge faster then the two-layered SNN with 6,400 excitatory neurons if both the networks were trained with the same learning rate. We find that the classification accuracy of the proposed models is lower than that achieved by the two-layered SNN because of the sparse recurrent inhibitory connections inside the liquid as explained below. When a test pattern is presented to the liquid, the neurons that learnt the corresponding pattern during training fire and only sparsely inhibit the remaining liquid neurons. This could potentially cause neurons that learnt different input classes but share common features with the presented test pattern to fire, leading to degradation in the classification accuracy. In order to precisely recognize a test pattern, it is important to attribute higher weight to the spike count of the correctly firing neurons and lower weight to the spike count of the incorrectly firing neurons. This can be accomplished by adding a readout layer and suitably adjusting the liquid to readout synaptic weights. We refer the readers to the Supplementary Material for performance characterization of SpiLinC with readout layer. Our results show that SpiLinC augmented with readout layer provides classification accuracy of 97.49% on the MNIST dataset and 97.29% on the TI46 digit subset.

**Table 6:** Classification accuracy of different SNN models (with similar audio pre-processing front-end) on 200 test samples from the TI46 speech corpus.

**SNN models**	**Network size**	**Training methodology**	**Accuracy (%)**
LSM (Verstraeten et al., 2005a)	1,200 liquid neurons	Supervised linear classifier	94.0
Liquid-SNN (our work)	1,600 liquid neurons	Unsupervised STDP	91.6
Two-liquid SpiLinC (our work)	2 × 800 liquid neurons	Unsupervised STDP	91.4

**Table 7:** Classification accuracy of different SNN models trained using unsupervised STDP on the MNIST dataset.

**SNN models**	**Network size**	**Number of synapses**	**Accuracy (%)**
Two-layered SNN (Diehl and Cook, 2015)	12,800 neurons	45,977,600	95
Liquid-SNN (our work)	12,800 liquid neurons	12,697,600	89.65
Four-liquid SpiLinC (our work)	4 × 3,200 liquid neurons	4,866,048	90.90

Finally, we note that the deep learning networks (Wan et al., 2013) have been shown to achieve 99.79% classification accuracy on the MNIST dataset while the Long Short-Term Memory (LSTM) Recurrent Neural Networks (RNNs) (Graves et al., 2004) provide 98% classification accuracy on the TI46 digit subset. Although the proposed liquid models yield lower classification accuracy than the deep learning networks and the LSTM-RNNs, they offer the following benefits with respect to computational efficiency and training complexity. First, the event-driven spike-based computing capability of the Liquid-SNN and SpiLinC naturally leads to improved computational efficiency than the deep learning networks including the binary networks (Hubara et al., 2016) that are data-driven and operate on continuous real-valued and discrete neuronal activations, respectively. Second, the deep learning networks and the LSTM-RNNs are respectively trained using error backpropagation (Rumelhart et al., 1986) and backpropagation-through-time (Werbos, 1990) algorithms, which are computationally expensive compared to the STDP-based localized training rule used in this work for the input to liquid synaptic weights. Last, the deep learning networks are iteratively trained on multiple presentations of the training dataset to minimize the training loss and achieve convergence. On the other hand, the proposed models are capable of achieving convergence with fewer training examples as evidenced by the four-liquid SpiLinC for MNIST digit recognition, which needed 64,000 training examples for convergence that roughly translates to single presentation of the training dataset. We note that meta-learning strategies (Hochreiter et al., 2001) have been proposed for LSTM-RNNs to learn quickly from fewer data samples by exploiting the internal memory in LSTM-RNNs. Recently, a new class of networks known as the memory-augmented networks (Santoro et al., 2016), where the networks are augmented with an external memory module, have been demonstrated for one-short learning or learning new information after a single presentation. Similar learning strategies, which either exploit the internal memory of a recurrently-connected liquid or incorporate an external memory module, can be used to improve the training efficacy of the proposed models.

## 5. Conclusion

In this work, we proposed Liquid-SNN consisting of input neurons sparsely connected to a randomly interlinked liquid for unsupervised speech and image recognition. We showed that adapting the input to liquid synaptic weights enables the neurons in a non-linear liquid to self-learn time-varying speech and static image patterns. We demonstrated the generality of our Liquid-SNN by training it to infer digit utterances from the TI46 speech corpus and handwritten MNIST digits. Such a general computing model, capable of processing different signal modalities using a uniform self-learning methodology, is highly desirable for neuromorphic hardware implementations. However, the Liquid-SNN suffers from scalability issues because of the need to grow the liquid for enhancing the classification accuracy. We proposed SpiLinC that is composed of an ensemble of smaller liquids trained in parallel to learn distinctive input features. The ability of SpiLinC to recognize an input by identifying low-level features improves the classification accuracy. SpiLinC, with adequately sized liquids for efficient feature learning, provided competitive accuracy with added sparsity in synaptic connectivity and faster training convergence across different application domains. Therefore, SpiLinC is a universal and scalable computing model that can be used to realize intelligent devices capable of real-time online learning.

## Author contributions

GS wrote the paper and performed the simulations. All authors helped with developing the concepts, conceiving the experiments, and writing the paper.

### Conflict of interest statement

The authors declare that the research was conducted in the absence of any commercial or financial relationships that could be construed as a potential conflict of interest.
