# Supplementary material for: SpiLinC: Spiking Liquid-Ensemble Computing for Unsupervised Speech and Image Recognition
Source: Front Neurosci. 2018 Aug 23;12:524. doi: 10.3389/fnins.2018.00524 (PMC6116788; doi:10.3389/fnins.2018.00524)
Supplement: Supplementary file 1 [file Data_Sheet_1.pdf]

Supplementary Material: SpiLinC: Spiking Liquid-Ensemble Computing for Unsupervised Speech and Image Recognition

1 PERFORMANCE CHARACTERIZATION OF SPILINC WITH READOUT

We augment SpiLinC with softmax readout layer containing as many neurons as the number of classes for a given pattern recognition task, where each readout neuron is fully-connected to the liquid-excitatory neurons. For a given input pattern, we use the STDP-trained input to liquid-excitatory synaptic weights to estimate the spike count of the liquid-excitatory neurons over the simulation interval, which are subsequently fed to the softmax readout layer. We train the liquid to readout synaptic weights using the Adam optimizer (Kingma and Ba, 2014), which is a popular gradient-based supervised training algorithm for deep learning networks, and cross-entropy loss function. During inference, we present a test pattern to the liquid, feed the spike count of the liquid-excitatory neurons to the softmax readout layer, and predict the test pattern to belong to the class represented by the readout neuron with the highest activation. For TI46 speech recognition, we initially trained the readout layer of two-liquid SpiLinC containing 800 neurons per liquid with learning rate of 0.015 on smaller digit subset of the TI46 speech corpus containing 300 training samples and 200 test samples. We obtained classification accuracy of 97.5% after 2 presentations of the training subset, which is higher than an accuracy of 94% provided by the LSM (Verstraeten et al., 2005) containing 1200 neurons that is trained with linear classifier. Further, we trained the readout layer of two-liquid SpiLinC containing 1600 neurons per liquid on the entire digit subset of the TI46 speech corpus containing 1594 training samples and obtained classification accuracy of 97.29% on the testing subset of 2542 test samples after 4 training epochs. Each training epoch consists of 16 presentations of randomly selected mini-batch of 100 training samples per presentation. For MNIST digit recognition, we trained the readout layer of four-liquid SpiLinC containing 3200 neurons per liquid with learning rate of 0.0022. The classification accuracy was 97.14% after one training epoch and increased to 97.49% after 4 training epochs. Each training epoch consists of 300 presentations of randomly selected mini-batch of 200 training examples per presentation. The classification accuracy of the four-liquid SpiLinC with readout is comparable to SNNs that use unsupervised STDP for the input and hidden layers, and supervised algorithms for the readout (or output) layer as shown in Table S1.

REFERENCES

Ferré, P., Mamalet, F., and Thorpe, S. J. (2018). Unsupervised feature learning with winner-takes-all based stdp. *Frontiers in computational neuroscience* 12, 24

Table S1. Classification accuracy of different SNN models that use unsupervised STDP for the input and hidden layers, and supervised algorithms for the readout (or output) layer on the MNIST dataset.

SNN Models	Training Methodology	Accuracy
Convolutional SNN (Kheradpisheh et al., 2018)	Unsupervised STDP + SVM classifier	98.4%
Convolutional SNN (Ferré et al., 2018)	Unsupervised STDP + Backpropagation	98.49%
Four-liquid SpiLinC (our work)	Unsupervised STDP + Backpropagation	97.49%

Kheradpisheh, S. R., Ganjtabesh, M., Thorpe, S. J., and Masquelier, T. (2018). Stdp-based spiking deep convolutional neural networks for object recognition. *Neural Networks* 99, 56–67. doi:https://doi.org/10.1016/j.neunet.2017.12.005

Kingma, D. P. and Ba, J. (2014). Adam: A method for stochastic optimization. *arXiv preprint arXiv:1412.6980*

Verstraeten, D., Schrauwen, B., and Stroobandt, D. (2005). Isolated word recognition using a liquid state machine. In *Proceedings of the 13th European Symposium on Artificial Neural Networks (ESANN)* (Bruges, Belgium), 435–440
